# Postmastectomy radiotherapy in breast reconstruction: Current controversies and trends

**DOI:** 10.1002/cai2.104

**Published:** 2024-01-07

**Authors:** Honghong Zhang, Dandan Song, Liangxi Xie, Ning Zhan, Wenjia Xie, Jianming Zhang

**Affiliations:** ^1^ Department of Radiation Oncology, Xiang'an Hospital of Xiamen University, Cancer Research Center, School of Medicine Xiamen University, Xiang'an Xiamen Fujian China; ^2^ Fujian Provincial Key Laboratory of Intelligent Identification and Control of Complex Dynamic System, Quanzhou Institute of Equipment Manufacturing, Haixi Institutes Chinese Academy of Sciences Quanzhou Fujian China

**Keywords:** breast reconstruction, postmastectomy radiotherapy, radiotherapy techniques

## Abstract

Breast cancer is the most common cancer among women worldwide. Postmastectomy radiotherapy (PMRT) is an essential component of combined therapy for early‐stage, high‐risk breast cancer. Breast reconstruction (BR) is often considered for patients with breast cancer who have undergone mastectomy. There has been a considerable amount of discussion about the optimal approach to combining PMRT with BR in the treatment of breast cancer. PMRT may increase the risk of complications and prevent good aesthetic results after BR, while BR may increase the complexity of PMRT and the radiation dose to surrounding normal tissues. The purpose of this review is to give a broad overview and summary of the current controversies and trends in PMRT and BR in the context of the most recent literature available.

## Graphical abstract

Postmastectomy radiotherapy in breast reconstruction: current controversies and trends in types of breast reconstruction, timing of radiotherapy, and radiotherapy techniques.

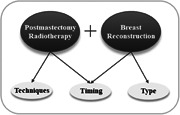

**Abbreviations**
ATR: autologous tissue (flap) reconstruction
BR: breast reconstruction
CI: confidence interval
IMN: internal mammary node
IMRT: intensity‐modulated radiation therapy
IPI: irradiation of permanent implant
IR: implant reconstruction
ITE: irradiation of tissue expander
NA: not applicable
OR: odds ratio
PMRT: postmastectomy radiotherapy
V20: total volume of lung receiving a dose of ≥20 Gy
VMAT: volumetric modulated arc therapy

## 1 INTRODUCTION

Breast cancer has overtaken other cancers as the most frequently diagnosed malignant disease, and is the leading cause of cancer mortality in women globally, with an estimated 2.3 million new cases per year [1]. Approximately 70%–80% of patients with early‐stage nonmetastatic breast cancer can now be cured [2]. The cure rate has been increasing as a result of combined therapy, with radiotherapy playing a significant role. Postmastectomy radiotherapy (PMRT) is important for high‐risk patients and has been confirmed to improve local tumor control and the survival rate [3]. Women who have undergone mastectomy, especially younger women, often require breast reconstruction (BR). The proportion of patients undergoing reconstruction after mastectomy in the US increased significantly from 26.94% to 43.30% between 2005 and 2014 (*p* < 0.01) [4]. Now that BR is being increasingly performed, radiation oncologists must deal with the difficulty of integrating PMRT with BR. Although PMRT may reduce the rate of local tumor recurrence, it may also increase the incidence of complications and prevent good aesthetic results from BR. Furthermore, although BR improves patient satisfaction, it also increases the complexity of PMRT and the irradiation dose to the surrounding normal tissues. There are still no standards regarding the optimal type of reconstruction for patients who require PMRT, the optimal timing of BR, or how to optimize radiotherapy techniques for patients undergoing BR. This review discusses and summarizes these issues in light of the latest literature.

## 2 SELECTION OF TYPE OF BR

Depending on the reconstruction material used, BR can be divided into autologous tissue (flap) reconstruction (ATR), implant reconstruction (IR), and reconstruction with a combination of two materials (e.g., latissimus dorsi muscle combined with an implant) [5]. ATR mainly uses a transverse rectus abdominus myocutaneous flap, latissimus dorsi flap, or other tissue flaps to reconstruct the breast, while IR mainly uses prostheses for reconstruction. There are several advantages and disadvantages to both ATR and IR. Although ATR typically requires only one procedure and creates a natural contour and soft texture, it is more invasive and difficult to perform and necessitates a longer recovery period. While IR is less complicated and leaves no scars in the donor area, it typically requires two or more operations and has risks, such as capsular contracture and implant rupture [5]. The overall condition of the breast, the patient's preferences, and the type of postoperative adjuvant therapy should be considered when selecting the type of BR.

The strength of evidence for most of the studies that have directly compared ATR with IR has been low to moderate. A 2011 meta‐analysis that compared these two types of reconstructions found that the postoperative complication rate was lower with ATR than with IR when radiation was administered following mastectomy (odds ratio [OR] = 0.20, 95% confidence interval [CI]: 0.11–0.39) [6]. This finding is consistent with the results of the 2018 prospective multicenter Mastectomy Reconstruction Outcomes Consortium cohort study, which included 643 patients who underwent ATR and 1604 who underwent IR at any of 11 participating institutions [7]. In 622 patients who received radiotherapy, the 2‐year complication rate was lower for ATR than for IR (OR = 0.47, 95% CI: 0.27–0.82, *p* = 0.007); furthermore, ATR had a lower reconstruction failure rate (1.0% vs. 18.7%) and a higher breast satisfaction score (63.5 vs. 47.7). Similarly, 2 years after surgery, radiation was associated with a 2.64‐fold higher risk of complications in the IR group (95% CI: 1.77–3.94, *p* < 0.001) but with an equal risk in the ATR group (OR = 1.12, 95% CI 0.66–1.92, *p* = 0.67). A 2022 meta‐analysis of 40 studies found clinically significant improvements in breast satisfaction and sexual well‐being in patients who underwent ATR in comparison with those who underwent IR [8]. While patients who underwent ATR had a higher risk of venous thromboembolism, those who underwent IR had a higher risk of reconstruction failure and seroma at follow‐up 1.5–4 years later.

Although all the abovementioned studies found that IR had higher reconstruction failure and complication rates than ATR when combined with PMRT, it is important to note that the strength of evidence in most of the studies was relatively low. In reality, IR is performed more frequently. According to a review of the US MarketScan database, the reconstruction rate increased from 46% to 63% between 1998 and 2007, with the increase being driven predominantly by the increase in IR [9]. In 2019, Hamann et al. [10] found that 84.6% of patients who underwent both PMRT and IR reported being willing to undergo the same reconstruction technique again. Furthermore, implant‐based second‐stage reconstruction may preserve the option of subsequent reconstruction, including ATR.

## 3 TIMING OF PMRT IN PATIENTS UNDERGOING BR

**Figure 1:**
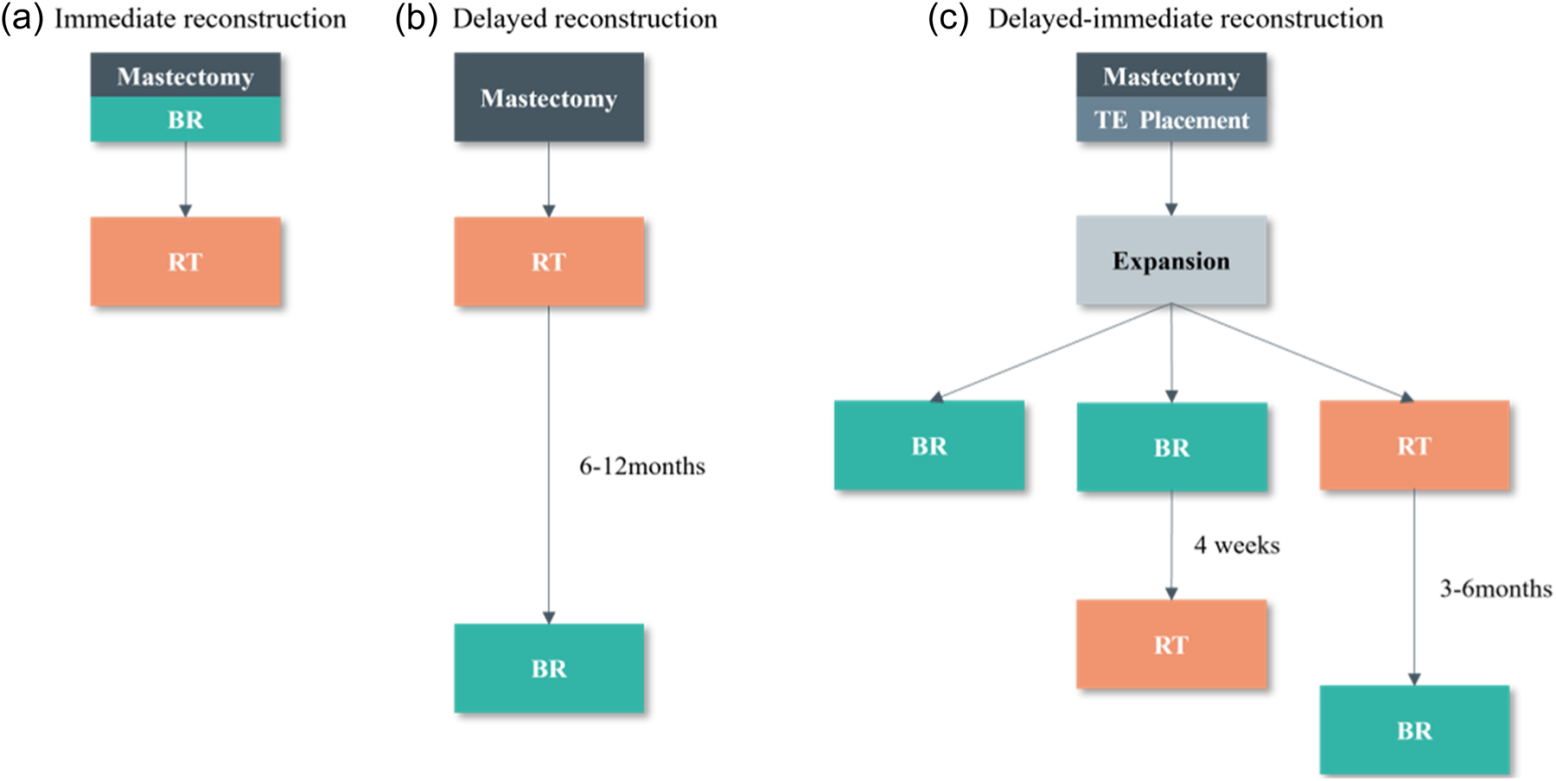
Schematic diagram showing the timing of immediate, delayed, and delayed‐immediate breast reconstruction. BR, breast reconstruction; RT, radiotherapy; TE, tissue expander.

BR is classified as immediate, delayed, or delayed‐immediate (Figure 1) depending on the timing of reconstruction [11]. In immediate BR, reconstruction is carried out simultaneously with total mastectomy in a single procedure under general anesthesia. The benefits of immediate BR include the potential to preserve critical anatomical elements, such as the breast skin and even the nipple and areola, lower surgery costs, and sparing patients the anguish of losing a breast. Delayed BR refers to BR that is performed a few months or years after a total mastectomy. Patients who have lost their breasts without immediate BR are more eager to undergo BR. If it is not clear whether radiotherapy is required during mastectomy, a tissue expander can be implanted first and then replaced with a permanent breast prosthesis or autologous tissue during BR according to the postoperative pathological results. This two‐stage BR procedure is called delayed‐immediate BR. More research is needed to clarify the optimal timing of PMRT in patients undergoing BR.

### 3.1 Timing of radiotherapy in patients undergoing ATR

In patients who require both PMRT and ATR, complications of reconstruction and aesthetic outcomes are the main considerations when choosing between immediate BR and delayed BR. In 2001, researchers at the MD Anderson Cancer Center compared the risk of complications between 32 patients who underwent immediate BR and 70 who underwent delayed BR. The follow‐up duration was 3 years in the immediate BR group and 5 years in the delayed BR group. While the incidence of early complications did not differ significantly between the two groups, patients who underwent immediate BR had a significantly higher incidence of late complications, including contracture, volume loss, and fat necrosis, than those who underwent delayed BR (87.5% vs. 8.6%, *p* < 0.001). Twenty‐eight percent of patients in the immediate BR group required additional flap surgery to correct flap shrinkage and severe flap contraction [12]. Therefore, most plastic surgeons prefer delayed BR for patients who require PMRT.

Immediate reconstruction using autologous tissue is strongly supported by the emergence of new encouraging data. A 2013 meta‐analysis showed that the outcomes were more satisfactory in patients who underwent immediate ATR before PMRT than in those who underwent delayed ATR after PMRT. There was no significant between‐group difference in the incidence of fat necrosis (*p* = 0.25) or the overall incidence of complications (*p* = 0.53). However, patients who underwent delayed BR were significantly less likely to require revision surgery (*p* = 0.001) [13]. This is consistent with the findings of two systematic reviews published in 2014. Both reviews reported similar complication rates in the two groups but significantly higher rates of revision surgery in patients who underwent immediate BR before PMRT [14, 15]. In 2017, the Mastectomy Reconstruction Outcomes Consortium also investigated this issue in a prospective multicohort study [16]. A total of 175 patients who underwent ATR were included, and no significant difference in the 1‐year complication rate was found between patients who underwent PMRT after immediate BR and those who underwent delayed BR (25.9% vs. 26.9%, *p* = 0.54). Although the breast, mental health, and sexual health satisfaction scores were significantly lower in patients in the delayed BR group than in those in the immediate BR group, the two groups had comparable satisfaction scores at 1–2 years after reconstruction. Similar findings were reported in a prospective study that evaluated the effect of radiation on immediate ATR [17]. There was no increase in the incidence of fat necrosis, fat necrosectomy, wound healing, or volume enhancement surgery in the radiation group in comparison with the group that did not receive radiation. A 2021 network meta‐analysis of 16 studies that compared various reconstruction times and types found no significant difference in the incidence of reconstruction failure (OR = 0.60, 95% CI: 0.11–3.32) or that of infection between immediate BR and delayed BR [18]. However, patients who underwent immediate BR had a higher risk of developing contracture than those who underwent delayed BR (OR = 121.03, 95% CI: 19.24–761.56). In general, recent studies have shown that immediate ATR is well tolerated following radiotherapy; therefore, it is regarded as an alternative for patients who require PMRT.

In 2011, Fosnot et al. [19] sought to determine the best time for delayed BR from the perspective of vascular complications in a study that included a total of 1025 reconstructed flaps, of which 226 were within the irradiated field. They found that the incidence of vascular complications was higher in the group that received radiation before delayed BR than in the group that did not (17.3% vs. 9.6%, *p* = 0.001) and that the risk of occurrence decreased with a longer duration of PMRT, suggesting that delayed BR after 3 months of radiotherapy may be prudent. In the same year, researchers at MD Anderson Cancer Center found that the incidence of repeat surgery was significantly higher in patients who underwent BR sooner than 12 months after PMRT than in those who underwent BR later than 12 months after PMRT (15% vs. 5%, *p* = 0.022) [20]. All flap losses occurred in patients who underwent BR sooner than 12 months after PMRT. Further analysis revealed that repeat surgery was required primarily in patients who underwent BR sooner than 6 months after PMRT. There was no significant difference in the incidence of microvascular thrombosis, infection, fat necrosis, partial flap loss, or wound dehiscence between the two groups. However, in another study, Momoh et al. [21] reported that there was no significant difference in the incidence of complications between patients who underwent BR sooner than 12 months after PMRT and those who underwent BR later and came to the same conclusion regarding these patients when BR was performed sooner or later than 6 months after PMRT. A recently published retrospective study investigated 303 patients who underwent delayed microvascular BR after PMRT [22]. The patients were divided into three groups according to whether they underwent reconstruction at 0–12 months, 12–18 months, or 18–50 months after PMRT. The incidence of major complications was not significantly different among the three groups (*p* = 0.57). Most of the major complications occurred 2–6 months after PMRT; however, this finding did not reach statistical significance. Based on the available evidence, the optimal waiting period between PMRT and delayed BR remains unclear. However, it is obvious that the longer the interval, the better the repair of skin damage after radiotherapy and the lower the risk of repair‐related complications after BR.

### 3.2 Timing of radiotherapy in patients undergoing IR

IR can be divided into single‐ or two‐stage reconstruction. Placing a permanent implant at the time of mastectomy is known as single‐stage reconstruction. In the two‐stage reconstruction, a tissue expander is placed under the skin and chest wall muscles at the time of mastectomy and replaced with a permanent prosthesis after the skin has expanded sufficiently [23]. Two‐stage expander prosthesis reconstruction is the most widely used technique in patients who require both PMRT and IR.

The choice of optimal timing of radiotherapy remains difficult in two‐stage reconstruction; depending on timing, it can be divided into irradiation of tissue expander (ITE) and irradiation of permanent implant (IPI). Many studies have indicated that radiotherapy increases the risks of reconstruction failure and periosteal contracture, regardless of whether the expander or permanent implant is irradiated [7, 24, 25]. However, the results of these studies are not consistent. The results of studies that compared ITE and IPI in ≥150 cases are summarized in Table 1. Nava et al. [26] found a significantly higher reconstruction failure rate in the ITE group than in the IPI group (40% vs. 6.4%, *p* < 0.0001) and a higher risk of Baker grade IV capsular contracture in the ITE group (13.3% vs. 10.1%). The findings of Nava et al. are consistent with those of Cordeiro et al.; however, Cordeiro et al. [27] found that the incidence of grade III–IV capsular contracture was much higher in the IPI group than in the ITE group (grade III, 44.6% vs. 15.9%; grade IV, 6.3% vs. 1.22%). Naoum et al. investigated 309 patients who underwent PMRT and two‐stage IR at Massachusetts General Hospital in 2022 [28]. Adjusted multivariable analysis showed no significant difference in the incidence of capsular contracture between the ITE and IPI groups (13.6% vs. 19.1%) but found a significantly higher overall reconstruction failure rate in the ITE group (39.5% vs. 31.5%, OR = 2.11, *p* = 0.02). Three meta‐analyses published between 2017 and 2022 yielded consistent results in terms of capsular contracture, namely, that its incidence was significantly higher in the IPI group than in the ITE group [31, 32, 33]. We conclude that ITE appears to be associated with a higher rate of reconstruction failure, whereas IPI seems to be associated with a higher rate of capsular contracture. The higher reconstruction failure rate in the ITE group may reflect the inability of tissue expanders to fully expand while the lower capsular contracture rate in the ITE group may be the result of the release of scar tissue during replacement surgery.

**Table 1:** Rates of reconstruction failure and capsular contracture for ITE versus IPI.

				Numbers of patients	Median follow‐up (months)	Rate of Reconstruction Failure(%)		Rate of Capsular Contracture(%)	
Reference	First Author	Year	Type of Study	ITE versus IPI	ITE versus IPI	ITE versus IPI	*p* value	ITE versus IPI	*p* value
Ref. [26]	Nava	2011	Prospective	50 versus 109	50 versus 50	40 versus 6.4	＜0.0001	Grade IV: 13.3 versus 10.1	NA
Ref. [27]	Cordeiro	2015	Prospective	94 versus 210	30.1 versus 40.3	32 versus 16.4*	<0.01	Grade III–IV: 17.1 versus 50.9	<0.01
Ref. [28]	Naoum	2022	Retrospective	220 versus 89	84.3 versus 45.5	39.5 versus 31.5	0.02	13.6 versus 19.1	0.81
Ref. [29]	Yoon	2020	Prospective	237 versus 80	NA	NA	0.488	NA	0.615
Ref. [30]	Santosa	2016	Prospective	104 versus 46	16.8 versus 14.1	11.5 versus 8.7	0.9	2.9 versus 2.2	0.804

*Note*: *: Six‐year predicted failure rates.

Abbreviations: ITE, irradiation of tissue expander; IPI, irradiation of permanent implant; NA, not applicable.

However, two prospective multicentre studies from the Mastectomy Reconstruction Outcomes Consortium have found that the timing of radiation intervention may not be a major predictor of complications. Yoon et al. [29] and Santosa et al. [30] found no significant difference in the overall complication rate, major complication rate, or reconstruction failure rate between 150 patients in an ITE group who underwent implant‐based two‐stage reconstruction and 317 in an IPI group who underwent the same procedure. However, it is important to note that both studies had a short follow‐up duration, and the rate of capsular contracture may have differed with a longer follow‐up duration [27].

In addition to follow‐up duration, differences in radiation techniques, radiation doses, definition of complications, reconstructive risk factors, and the methods used for statistical analysis may affect the incidence of reconstruction complications. Considering that the risk of severe capsular contracture associated with PMRT is higher than that associated with failed reconstruction, some physicians have suggested that ITE should be performed and that those with poor results after ITE be considered for conversion to ATR [34]. When performing two‐stage reconstruction and PMRT, physicians should fully inform patients about the impact of radiotherapy on BR so that patients can make appropriate decisions.

When to implant a permanent prosthesis in patients who undergo expander irradiation is also a topic worthy of discussion. A 2012 study of 88 patients who underwent radiotherapy and two‐stage reconstruction found that the reconstruction failure rate was significantly lower in those who underwent exchange surgery later than 6 months after radiotherapy than in those who underwent the procedure sooner than 6 months after radiotherapy (7.7% vs. 22.4%, *p* = 0.036) [35]. Similarly, in 2015, Fowble et al. [36] found that a short interval between exchange surgery and radiotherapy increased the reconstruction failure rate. When the interval was less than 3 months, the reconstruction failure rate increased by 6.375‐fold. Lentz et al. [37] did not find a significant difference in the overall complication rate or the reconstruction failure rate between an early exchange group (<4 months after radiotherapy) and a late exchange group (>4 months after radiotherapy). However, the early exchange group appeared to have a higher rate of infection and the late exchange group had a higher rate of capsular contracture. In 2022, Kim et al. [38] investigated the optimal timing of exchange to reduce the incidence of short‐term (49 days) and long‐term (2 years) complications and found that placing a permanent implant 131 days after radiotherapy reduced the incidence of short‐term complications. At present, the optimal timing of the exchange is still uncertain. However, in general, it should be done when the skin has fully recovered after radiotherapy.

## 4 DEVELOPMENT OF RADIOTHERAPY TECHNIQUES FOR BR

From the radiation oncologist's perspective, the goal of a PMRT plan is to achieve optimal coverage of the target volume while minimizing the irradiation dose to normal tissues. Previous studies have shown significant reductions in chest wall coverage, internal mammary node (IMN) coverage, and avoidance of the heart and lung in patients who undergo immediate BR and PMRT in comparison with patients who undergo PMRT without immediate BR, especially in those with left‐sided lesions [39, 40]. However, more recent studies have reached different conclusions. In 2013, Chung et al. [41] found that it was feasible to deliver an adequate dose to the reconstructed breast using standard field arrangements regardless of the laterality of the treatment plan or the type of reconstruction but mentioned that irradiation of the IMN may increase the dose to the heart. In 2012, Ohri et al. [42] analyzed the impact of BR on radiotherapy plans from the perspective of the dose‐volume histogram. They found that the treatment technique, not BR, was the major determinant of the target coverage and the dose to normal tissues. However, irradiation of the IMN significantly increased doses to the heart and lungs (all *p* < 0.05). Irradiation of the IMN is clearly a key factor in determining whether PMRT can achieve the best curative effect. Fortunately, other studies have demonstrated that BR does not affect the quality of PMRT plans even with IMN irradiation. A retrospective study by Ben‐David et al. [43] evaluated the quality of radiotherapy plans in 29 patients who underwent immediate BR with implants. Two separate radiotherapy plans were designed for each patient based on the inclusion or exclusion of IMN. Comparing the two plans, they found that good coverage of the reconstructed breast (both ≥96.9%) was achieved with or without the inclusion of IMN, while heart and lung doses were low (the mean dose to the heart was <1.56 Gy and the mean total volume of lung receiving a dose of ≥20 Gy [V20] fluctuated between 13.8% and 19.47%). In 41 patients who underwent immediate breast expander reconstruction and PMRT, Koutcher et al. [44] found that the mean lung V20 was 13% (range, 3–23), and the mean dose to the heart was 2.81 Gy (range, 0.53–9.60). Patients with left‐sided lesions who underwent IMN irradiation had a mean lung V20 of 18% and a mean dose of 8.04 Gy to the heart. Although IMN irradiation increases the doses to the lungs and heart, the doses are within acceptable ranges.

As awareness of the risk factors for breast cancer has increased, so has the need for bilateral mastectomy and bilateral BR [45, 46]. This also raises the question for radiation oncologists of whether contralateral/bilateral BR makes it more difficult to administer radiation therapy. As early as 2014, Ho et al. [47] found that bilateral IR did not affect the quality of postoperative radiation plans. Their study included 197 patients who underwent radiation therapy and BR, 51% of whom underwent unilateral reconstruction and 49% underwent bilateral reconstruction. Chest wall coverage was good in up to 90% of patients in their entire cohort. Multivariate analysis found that the presence of contralateral implants did not result in higher irradiation doses to the heart or lungs (*p* = 0.54). However, it should be noted that 14% of patients who underwent bilateral reconstruction still received radiation with contralateral implants. Furthermore, Jagsi et al. [7] found that bilateral reconstruction was a risk factor for breast complications at 1 and 2 years, postoperatively. These findings suggest that we need to find ways to reduce irradiation to contralateral implants. There are few studies on this topic, and the current practice is to reduce irradiation to the contralateral implant or increase coverage of the IMN by moving the contralateral implant away from the treatment area or reducing the volume of the tissue expander [47, 48, 49].

PMRT has undergone a transition from two‐dimensional conventional and three‐dimensional conformal techniques to newer techniques, such as intensity‐modulated radiation therapy (IMRT), volumetric modulated arc therapy (VMAT), and proton radiation therapy [50, 51]. These three techniques have been shown to improve the quality of radiotherapy plans for patients undergoing BR [52, 53]. Compared with three‐dimensional conformal radiation therapy, IMRT and VMAT can deliver different doses to different target areas of the chest wall and lymph nodes, which can improve the uniformity of dose distribution in the target area and avoid high doses to the heart and lungs [54, 55]. VMAT has been shown to significantly reduce doses to the lung, heart, and contralateral breast/implant in patients with left‐sided breast cancer when combined with deep inspiration breath‐hold [56, 57]. However, breast radiotherapy with IMRT or VMAT increases exposure to large low‐dose areas (≤5 Gy) in the chest, which may increase the incidence of radiation‐related secondary tumors [58]. Further studies are required to determine the long‐term complications of IMRT and VMAT. Compared with photon therapy, proton therapy, as a result of its unique physical and biological properties, can significantly reduce the radiation dose to normal tissues around the tumor, such as the heart and lungs, without reducing the dose to the tumor. Lower radiation doses to the heart and lungs are associated with lower risks of late cardiac events and secondary primary tumors [59, 60, 61, 62]. Furthermore, proton therapy can be used to reduce the dose to surrounding normal tissue and avoid poor clinical target coverage in patients with IMN irradiation or left‐sided breast cancer [63, 64, 65].

Different radiation modalities, fractionations, and the use of a local boost or bolus may be crucial determinants of the ultimate cosmetic result in women undergoing BR. In patients undergoing mastectomy and BR, conventional fractionated radiation is typically administered at the recommended dose of 45.0–50.4 Gy in 25–28 fractions. Some studies have shown that hypofractionated radiotherapy after mastectomy is non‐inferior to conventional fractionated radiotherapy, which has led to increased interest in hypofractionated radiotherapy in BR [66]. A retrospective study in South Korea published in 2022 compared the impact of hypofractionated radiotherapy (2.4–2.7 Gy/fraction) with that of conventional fractionated radiotherapy (1.8–2.0 Gy/fraction) in 396 patients undergoing BR [67]. The median follow‐up duration was 35.3 months. The median dose to the whole breast or chest wall was 44.3 Gy (range, 40.5–48.6) for hypofractionated radiotherapy and 50.3 Gy (range, 50–66) for conventional radiotherapy. Hypofractionated radiotherapy appeared to be comparable with conventional fractionated radiotherapy in terms of the incidence of breast‐related complications in patients who underwent any type of BR. However, prospective studies are needed to confirm this finding. Khan et al. [68] enrolled 67 patients in a prospective study, 61% of whom underwent BR. Patients received hypofractionated radiotherapy consisting of a total dose of 36.63 Gy in 11 fractions. Some patients received additional local boosts of 4 fractions of 3.33 Gy. The median follow‐up duration was 32 months. No serious toxicity was found, and the incidence of reconstruction‐related complications was 32%, which is consistent with the findings of previous studies [69, 70]. Two prospective, randomized, controlled Phase III clinical studies comparing hypofractionated radiotherapy (42.56 Gy in 16 fractions) and conventional fractionated radiotherapy in patients undergoing BR are currently under way (NCT03414970 and NCT03422003). In theory, it makes sense to deliver higher doses to areas with a higher risk of local recurrence, such as radiation using local boosts or boluses in high‐risk areas, but there may be an increase in the incidence of reconstruction complications. Naoum et al. [71] retrospectively investigated the effect of a chest wall boost on the incidence of reconstruction complications and the rate of disease control in 746 patients who underwent PMRT and BR; 379 received a chest wall boost and 367 did not. They found that radiation with a chest wall boost was associated with an increased risk of IR failure. Furthermore, administration of chest wall boost radiotherapy did not improve the local tumor control rate, even in high‐risk patients. These findings suggest that omitting a chest wall boost in patients undergoing PMRT may improve the cosmetic outcome of BR without compromising local tumor control. This finding is consistent with the result of a single‐center retrospective study reported in 2018 American Society for Radiation Oncology. The study included 730 patients who underwent BR between 1997 and 2001, of whom 43.2% received PMRT with a chest wall boost and 56.7% received PMRT without a boost. The respective median follow‐up durations were 62 months and 75 months. The investigators found that the absence of a chest wall boost did not reduce the local tumor control rate but improved the outcome of BR. In the presence of tissue expanders or flaps, there is still considerable disagreement among US radiation oncologists about whether to use a bolus [72]. In patients with skin involvement, a bolus is usually preferred for skin coverage [73]. There are fewer studies on whether a bolus affects BR. A recently reported small retrospective study that included 25 patients who underwent two‐stage BR and received a bolus in PMRT found that adding a bolus significantly increased the reconstruction failure and infection rates in the first stage of BR [74]. Large randomized controlled studies are needed to clarify the effect of boluses on BR.

## 5 CONCLUSION

PMRT and BR have become important components of treatment in patients with high‐risk breast cancer after mastectomy. Existing studies do not clarify the optimal type of BR or the optimal timing of PMRT. ATR appears to be more resistant to radiotherapy than IR, but IR preserves the option of choosing another type of reconstruction later. The longer the interval between PMRT and BR, whether autologous tissue‐based or implant‐based, the lower the incidence of reconstruction‐related complications. More prospective studies are expected to provide more definitive answers in these areas. At present, the choice of BR type and the timing of PMRT should be carefully considered based on the patient's tumor status, physical condition, cosmetic requirements, quality of life, and other factors to minimize tumor recurrence and reconstruction complication rates and improve patient satisfaction.

## AUTHOR CONTRIBUTIONS

**Honghong Zhang**: Writing—original draft (equal); writing—review & editing (equal). **Dandan Song**: Writing—original draft (equal); writing—review & editing (equal). **Liangxi Xie**: Conceptualization (supporting); writing—review & editing (equal). **Ning Zhan**: Writing—review & editing (supporting). **Wenjia Xie**: Conceptualization (equal); writing—review & editing (equal). **Jianming Zhang**: Conceptualization (equal); funding acquisition (lead); supervision (lead).

## CONFLICT OF INTEREST STATEMENT

The authors declare no conflict of interest.

## ETHICS STATEMENT

Not applicable.

## INFORMED CONSENT

Not applicable.

## Data Availability

Data sharing is not applicable — no new data are generated, or the article describes entirely theoretical research.
